# Less for Better: A View Filter-Driven Graph Representation Fusion Network

**DOI:** 10.3390/e28010026

**Published:** 2025-12-24

**Authors:** Yue Wang, Xibei Yang, Keyu Liu, Qihang Guo, Xun Wang

**Affiliations:** 1 School of Computer, Jiangsu University of Science and Technology, Zhenjiang 212003, China; 2 School of Economics and Management, Jiangsu University of Science and Technology, Zhenjiang 212100, China

**Keywords:** multi-viewlearning, graph representation fusion, graph neural networks, graph entropy

## Abstract

Multi-view learning has recently gained considerable attention in graph representation learning as it enables the fusion of complementary information from multiple views to enhance representation quality. However, most existing studies neglect that irrelevant views may introduce noise and negatively affect representation quality. To address the issue, we propose a novel multi-view representation learning framework called a **Vi**ew **Fi**lter-driven graph representation fusion network, named **ViFi**. Following the “less for better” principle, the framework focuses on filtering informative views while discarding irrelevant ones. Specifically, an entropy-based adaptive view filter was designed to dynamically filter the most informative views by evaluating their feature–topology entropy characteristics, aiming to not only reduce irrelevance among views but also enhance their complementarity. In addition, to promote more effective fusion of informative views, we propose an optimized fusion mechanism that leverages the filtered views to identify the optimal integration strategy using a novel information gain function. Through extensive experiments on classification and clustering tasks, ViFi demonstrates clear performance advantages over existing state-of-the-art approaches.

## 1. Introduction

Graph-structured data increasingly exhibit multi-view characteristics with the development of graph representation learning. For instance, social networks encompass multiple views, including user profiles, interaction histories, and content preferences. Multi-view learning is crucial as it integrates diverse perspectives, enhancing model performance on downstream tasks and enabling more reliable analysis of complex data [1,2]. Multi-view learning has undergone significant advances across diverse domains, including computer vision [3], natural language processing [4], and bioinformatics [5].

Recently, owing to the powerful capability of graph neural networks (GNNs) in capturing complex structural relationships and semantic information [6,7,8], the collaboration between multi-view learning and GNNs facilitates the extraction of view-specific representations and the discovery of inter-view correlations [9,10,11]. This collaboration enables multi-view GNNs to fully exploit complementary information distributed across views, leading to their promising performance in various real-world applications [12,13]. For example, in recommendation systems, multi-view GNNs fuse heterogeneous user–item interactions, achieving excellent recommendation accuracy.

**Figure 1:**
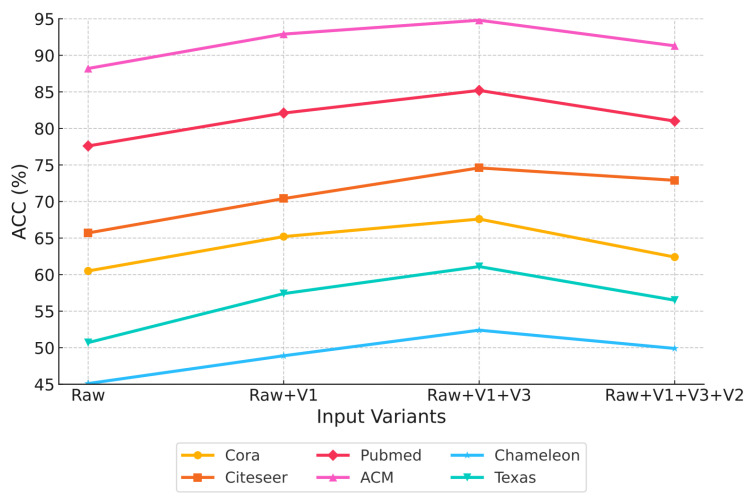
Validating the necessity of view filtering. As the number of views increases, the classification accuracy (ACC%) reaches a peak and subsequently declines, indicating that not all views contribute positively to model performance.

Currently, to enhance the effectiveness of multi-view fusion in representation learning, existing approaches typically employ attention [14], gating [15], alignment [16], or fusion modules [17] to jointly integrate information from all views. These methods assign weights or scores to each view and aggregate views accordingly, allowing models to emphasize informative views while suppressing irrelevant ones. However, these approaches aggregate all views based on the assumption that all views contribute positively to the learning process. We argue that this assumption is not always valid; thereby, we conducted a preliminary experiment to examine its validity. To assess the contribution of each view, we measured its information content using information entropy [18]. A greedy strategy was applied, where the raw view (Raw) was sequentially integrated with other views (V1, V2, and V3) in descending order of entropy. As illustrated in Figure 1, the classification accuracy initially increases but subsequently declines as more views are added, indicating that irrelevant views may interfere with model performance. Therefore, it is vital to design an effective mechanism to identify and retain only the views that contribute positively to representation while filtering out irrelevant views that contribute negatively.

To address the above issue, we propose a novel multi-view representation learning framework called a view filter-driven graph representation fusion network, named ViFi. Unlike prior approaches that weight or aggregate all views, ViFi operates on a “less for better” principle, aiming to obtain superior representations by leveraging fewer yet more informative views.

Initially, an entropy-based adaptive view filter was designed to identify and retain only the most informative views in a multi-view learning system, filtering out irrelevant views. The filter quantifies each view’s information content through feature–topology entropy characteristics [19], which effectively reflect the uncertainty and diversity of feature and topology distributions, serving as an indicator of a view’s information richness. By maximizing an entropy-based objective function, the module adaptively determines the optimal number of views to retain, filtering most informative views while discarding irrelevant views. The objective is formulated to reward views with substantial feature–topology entropy while penalizing irrelevance, thereby promoting a compact subset that preserves maximal informational diversity. The filter further stabilizes the subsequent fusion process by providing a compact and informative set of views.

In addition, to promote more effective fusion of informative views, we propose an optimized fusion mechanism that is introduced after filtering to adaptively determine the optimal integration strategy which yields the most informative representations. A novel information gain function is proposed to evaluate candidate view groupings based on entropy balance and structural complementarity. Entropy balance is achieved by normalizing the entropy of each view matrix and computing an equilibrium degree, which encourages uniform information contribution and avoids interference in fusion. Structural complementarity is enforced by normalizing structural differences and defining an information gain term that favors the grouping of view matrices exhibiting significant topological diversity. By selecting the subset that maximizes overall group gain as the optimal integration strategy, this module achieves balanced and complementary fusion, thereby facilitating effective collaboration among views.

To conclude, the main contributions of this study are presented as follows:

(1) A novel multi-view representation learning framework is proposed, which systemically combines view filtering with optimized fusion to produce compact, informative multi-view graph representations.
(2) An entropy-based adaptive view filter was developed which evaluates view contribution through feature–topology entropy and dynamically retains the most informative views to reduce irrelevance and enhance complementarity.
(3) A novel information gain function was designed to evaluate the contribution of different view integration strategies and guide the selection of an optimal integration strategy that achieves entropy balance and structural complementarity, thereby strengthening inter-view collaboration.
(4) Based on comprehensive experiments on classification and clustering tasks, the proposed method consistently achieves superior performance over existing state-of-the-art approaches.

## 2. Related Work

### 2.1. Multi-View Learning

Multi-view learning is a machine learning paradigm that aims to exploit complementary information from different perspectives or sources of data. By integrating multiple views, it enables the construction of more comprehensive and informative representations, which often lead to improved performance in various learning tasks. Traditional multi-view learning methods can be divided into three major categories [20]: co-training-style algorithms [21,22], co-regularization-style algorithms [23] and margin-consistency-style algorithms [24,25]. For instance, Qiao et al. [26] proposed Deep Co-Training, which trains multiple networks as distinct views under adversarial perturbation conditions to maintain complementary and diverse view representations. Wang et al. [27] introduced Deep Canonically Correlated Autoencoders (DCCAE), combining Canonical Correlation Analysis (CCA) with autoencoder reconstruction to co-regularize representations across views. The hybrid objective offers a flexible way to align and denoise multi-view features. Mao et al. [28] proposed Soft-Margin-Consistency Multi-View MED (SMVMED), relaxing hard margin-equality constraints into a soft consistency principle to improve scalability while preserving discriminative power. However, these studies generally integrate all available views, overlooking the potential interference of irrelevant views in graph representation learning. Inspired by these works, we attempted to design a view-filtering mechanism that retains most informative views while discarding irrelevant views.

### 2.2. Multi-View Representation Fusion

As a critical component of multi-view learning, multi-view representation fusion is not a novel topic, and it has been extensively explored in a range of existing studies. Based on the time of multi-view fusion during the learning process, traditional works can be divided into two primary types [29]. (1) Early fusion integrates features from multiple views at the feature level before model training through typical methods such as feature concatenation, pooling-based integration, and convolutional fusion. For example, Kachole et al. [30] concatenated RGB and event-stream features at the feature level to construct a unified multi-view representation, which effectively enhanced view complementarity and improved segmentation accuracy. Wei et al. [31] aggregated per-view CNN descriptors by adaptive view pooling over a learned view-graph to obtain a unified global shape descriptor. Liang et al. [32] employed 1 × 1 convolutions for channel-wise fusion of LiDAR and camera features before joint detection, significantly improving multi-view 3D detection accuracy. (2) Late fusion aggregates the predictions from different views after training separate models for each view through methods such as score averaging, weighted voting, and meta-classifier stacking. For instance, Simonyan et al. [33] averaged softmax scores from spatial and temporal streams to obtain a single decision, which consistently outperformed either stream alone on multi-view action recognition. Su et al. [34] used weighted voting to aggregate the prediction scores from multiple views, enabling a more robust fusion that improved the reliability of biomarker identification. Wang et al. [35] employed a meta-classifier to integrate the cross-validated outputs of view-specific CNN classifiers, enabling stacking-based fusion that better exploited heterogeneous cues and enhanced overall prediction accuracy.

Building upon the above fusion strategies, LoGo-GNN [36] can be regarded as a hybrid fusion framework that integrates both early and late fusion principles through a local-to-global architecture. In this framework, the local module performs fusion by integrating multiple view-specific representations through predefined aggregation pathways, enabling neighborhood information from different local views to be jointly embedded. The global module further aligns these aggregated representations across views to ensure overall consistency.

Despite its effectiveness, existing fusion frameworks, including LoGo-GNN, generally rely on fixed fusion strategies and manually designed aggregation schemes, which limits their ability to adapt to heterogeneous graph structures and dynamically varying inter-view relationships. Such rigidity may hinder the effective exploitation of complementary information across views, especially in complex multi-view graph scenarios. Motivated by this gap, this study extends the local–global architecture by introducing an optimized fusion mechanism that dynamically selects appropriate integration strategies, thereby enhancing flexibility and representation quality across diverse views.

## 3. ViFi

### 3.1. Notation

We consider a graph G=(𝒱,E), where 𝒱 denotes the set of nodes and E represents the set of edges. The adjacency matrix A encodes the graph structure, with each entry indicating the presence or absence of an edge between a pair of nodes. The node-feature matrix X contains attribute information associated with nodes, where each row corresponds to a node and each column represents a feature dimension.

The *i*-th graph view is denoted by $M_{i}$, representing a specific structural or feature-based perspective of the graph. Let *S* denote the selected subset of views, with k=|S| indicating the number of selected high-quality views and $T_{3}$ representing the minimum number of views required for effective fusion.

For each view $M_{i}$, $H_{f}\left(M_{i}\right)$ denotes the feature entropy, which measures the uncertainty and diversity of node features within the view, while $H_{s}\left(M_{i}\right)$ denotes the topology entropy, which characterizes the structural complexity and connectivity distribution of the corresponding graph view. Their combination yields the view information score $I(M_{i})$, reflecting the overall informativeness of a view. A subset *S* is further evaluated by the subset score F(S), which assesses the collective quality of selected views.

To characterize complementarity between different views, the entropy balance $B_{ij}$ is used to measure the information equilibrium between view pairs, while the normalized structural difference $\Delta_{ij}$ quantifies their structural dissimilarity. The product of these two terms defines the pairwise gain $G(M_{i},M_{j})$, and aggregating pairwise gains over the subset *S* yields the group gain G(S). g(·) denotes a graph neural network (GNN) encoder used to learn node representations from the fused views. Finally, ϕ(·) denotes the size penalty function.

Table 1 summarizes the notations used in this paper.

### 3.2. Framework Overview

**Figure 2:**
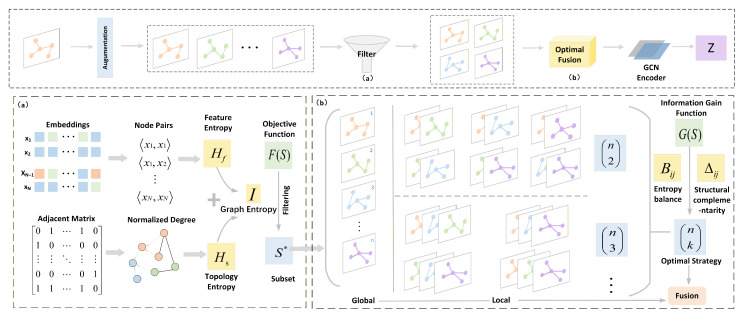
The framework of ViFi, where modules (**a**,**b**) represent the core components. Module (**a**) is the entropy-based adaptive view filter, and module (**b**) is the optimized fusion mechanism.

The overall structure of ViFi is illustrated in Figure 2. It mainly includes two modules. (1) The entropy-based adaptive view filter evaluates each view based on feature and topological entropy characteristics to adaptively filter those with the highest informational utility, thereby establishing a compact and diverse set of informative views. (2) The optimized fusion mechanism employs a novel information gain function, which is constructed from entropy balance and normalized structural difference across views. It effectively fuses the representations by dynamically selecting the optimal view integration strategy that maximizes the group gain.

### 3.3. Entropy-Based Adaptive View Filter

Multi-view learning often contains irrelevant views. For example, in recommendation graphs, views derived from clicks, purchases, and wishlists may overlap in signal, and sparsity varies across them. In molecular property prediction, 2D topology, 3D conformers, and substructure fingerprints provide complementary but uneven information. Passing all views downstream raises computation and risks over-fitting to noisy or irrelevant signals. Therefore, an entropy-based adaptive view filter was designed to address this issue by ranking and filtering only informative views and by adapting the number of retained views to the data.

We quantify the information content in each view by feature–topology entropy characteristics [19]: feature entropy and topology entropy. Feature entropy reflects uncertainty in node attributes under locality assumptions. Topology entropy captures higher-order topology using normalized structural statistics. Entropy is an appropriate metric here because it summarizes distributional uncertainty in a model-agnostic way and requires no task labels. Let a view $M_{i}=\left(A_{i},X_{i}\right)$. We define:

(1) $$H_{f}\left(M_{i}\right)\;=\;-\sum_{i}p_{i}^{\left(f\right)}\;\log p_{i}^{\left(f\right)},\;H_{s}\left(M_{i}\right)\;=\;-\sum_{i}p_{i}^{\left(s\right)}\;\log p_{i}^{\left(s\right)},$$

where $p^{\left(f\right)}$ and $p^{\left(s\right)}$ are normalized distributions induced by features and structure following Luo et al. [19]. We combine them as:

(2) $$I\left(M_{i}\right)\;=\;\alpha \;H_{f}\left(M_{i}\right)\;+\;\left(1-\alpha \right)\;H_{s}\left(M_{i}\right),$$

where α∈[0,1], and use $I(M_{i})$ only to drive the filter. Higher scores indicate that the view contains richer information and greater complementarity.

We now use entropy to filter a subset of views and to adaptively decide how many to keep. The key idea is to retain views with high information content while preventing the inclusion of an excessively large set that would reintroduce redundancy and increase computational cost. We score any candidate subset *S* by:

(3) $$F\left(S\right)=\frac{1}{\left|S\right|}\sum_{M_{i}\in S}I\left(M_{i}\right)-\mu \;\phi (|S|),\;\left|S\right|\geq T_{3}.$$

Here *S* denotes the filtered set of candidate views. $I(M_{i})$ is the per-view information score defined above. The parameter μ>0 controls the trade-off between information and complexity. The function ϕ(·) is an increasing size penalty, with common choices being $\phi (n)=\max(n-T_{3},0)$ and ϕ(n)=n. The constant $T_{3}=3$ imposes a minimum of three views to preserve basic multi-view complementarity. Maximizing F(S) yields a filter that keeps high-information views and discards redundant ones while automatically determining how many views to retain:

(4) $$S^{★}\;=\;\arg\max_{S}\;F\left(S\right).$$

This design makes the filter adaptive, reduces computation, and stabilizes downstream fusion by avoiding noisy or overlapping inputs.

The entropy-based adaptive view filter uses feature-topology entropy characteristics to quantify view information then maximizes *F*(*S*) to select an adaptive-size subset. The module filters out irrelevant views. It preserves complementary signal and yields a compact, informative set with less redundancy. This not only improves efficiency and robustness but also reduces the burden on the subsequent fusion stage. The specific implementation of the entropy-based adaptive view filter module follows the steps outlined in Algorithm 1.

**Algorithm 1** Entropy-based adaptive view filter**Input:** Graph views $\left\{M_{1},M_{2},\ldots ,M_{n}\right\}$, where each $M_{i}=\left(A_{i},X_{i}\right)$;
trade-off parameter α∈[0,1]; penalty coefficient μ>0;
minimum view count $T_{3}$; size penalty function g(·). **Output:** Filtered subset of views $S^{*}$.1:**for** each view $M_{i}$ **do**2:   Compute feature entropy $H_{f}\left(M_{i}\right)$ and topology entropy $H_{s}\left(M_{i}\right)$ via Equation (1);3:   Calculate feature–topology entropy as view content information via Equation (2);4:**end for**5:**Initialize:** candidate subset *S* with $\left|S\right|\geq T_{3}$;6:Compute the subset score F(S) by combining the average information score of views in *S* with a penalty term based on the subset size, as defined in Equation (3);7:Select the most informative subset $S^{*}$ by maximizing the subset score F(S) over all candidate subsets, following the process in Equation (4);8:**return** $S^{*}$.

### 3.4. Optimized Fusion Mechanism

**Figure 3:**
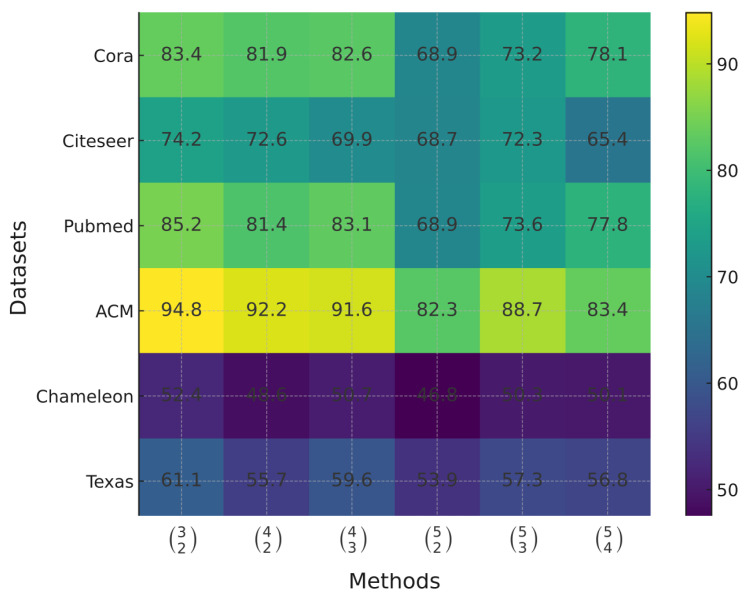
Heatmap of classification accuracy (ACC%) across six datasets and six view integration strategies. Brighter cells indicate higher accuracy. These patterns indicate that varying integration strategies lead to significant differences in classification performance.

Furthermore, to enable more effective fusion of informative views, we introduce a fusion mechanism based on a local-to-global framework, which promotes hierarchical integration of view-specific information and enhances overall representation consistency. To further investigate how different integration strategies influence representation quality, we conducted a preliminary experiment that evaluated the effect of different integration strategies on node classification accuracy across multiple datasets. The results shown in Figure 3 indicate that varying integration strategies lead to significant differences in classification performance. This observation motivates the need to adaptively identify the most effective view integration strategy for the fusion mechanism.

After obtaining a set of informative views, we need to decide which subset of the filtered views should be integrated so that their fused representation is maximally informative. The fusion objective is to balance the information contribution of each view and to exploit structural complementarity among them. To achieve this, we introduce a novel information gain function that scores candidate groups and selects the subset with the highest gain.

The goal of this module is to select an optimal subset *S* of filtered view matrices for joint fusion. Let $\left\{M_{1},\ldots ,M_{n}\right\}$ represent the candidate view matrices obtained from Module 1. We group these matrices into sets of size k≥2 since fusion requires at least two views. The objective is to find the grouping that maximizes the overall fusion score. Each group should maintain balanced information entropy across views and preserve structural complementarity. In this way, the fusion process avoids dominance by a single view and reduces irrelevance among similar structures.

Information gain is formulated as a multiplicative combination of entropy balance and structural difference to ensure that both aspects jointly influence the evaluation of view interactions. Entropy balance reflects the reliability and stability of the information carried by each view, while structural difference captures the degree of complementary topology between views. Using a multiplicative form links these two criteria in a way that prevents either from dominating the decision process. A view pair is considered valuable only when it provides both sufficiently balanced information and meaningful structural complementarity. This design avoids selecting views that are informative but redundant or structurally diverse but weak in information, thereby promoting more principled and discriminative fusion behavior.

To quantify these ideas, we start by defining a *pairwise gain* $G(M_{i},M_{j})$ between any two view matrices $M_{i}$ and $M_{j}$. First, let $H(M_{i})$ denote the Shannon entropy of matrix $M_{i}$, interpreted as the amount of information it contains. We define two factors:

(1) Entropy balance:

(5) $$B_{ij}=\frac{\min\;\left(H\left(M_{i}\right),\;H\left(M_{j}\right)\right)}{\max\;\left(H\left(M_{i}\right),\;H\left(M_{j}\right)\right)},$$

where $B_{ij}$ lies in [0,1]. When $H\left(M_{i}\right)\approx H\left(M_{j}\right)$, $B_{ij}$ is close to 1, indicating balanced information; if one entropy is much smaller than the other, $B_{ij}$ approaches 0, reflecting imbalance.

(2) Normalized structural difference:

(6) $$\Delta_{ij}\;=\;\frac{{∥M_{i}-M_{j}∥}_{F}}{{∥M_{i}∥}_{F}+{∥M_{j}∥}_{F}},$$

where ${∥\cdot ∥}_{F}$ is the Frobenius norm, defined as:

(7) $${∥M∥}_{F}=\sqrt{\sum_{p=1}^{m}\sum_{q=1}^{n}{\left|M_{pq}\right|}^{2}},$$

with $M\in R^{m\times n}$ representing a view matrix and $M_{pq}$ its (p,q)-th element. This measures how different the two matrices are, normalized by their magnitudes. The value $\Delta_{ij}\in \left[0,1\right]$ is 0 if $M_{i}=M_{j}$ (no structural difference) and approaches 1 as the difference grows large relative to its norms. This normalization makes $\Delta_{ij}$ comparable across different data scales.

The pairwise gain is then defined as the product of these two factors:

(8) $$G\left(M_{i},M_{j}\right)\;=\;B_{ij}\times \Delta_{ij}\;=\;\frac{\min\;\left(H\left(M_{i}\right),\;H\left(M_{j}\right)\right)}{\max\;\left(H\left(M_{i}\right),\;H\left(M_{j}\right)\right)}\cdot \frac{{∥M_{i}-M_{j}∥}_{F}}{{∥M_{i}∥}_{F}+{∥M_{j}∥}_{F}}.$$

According to this construction, $G(M_{i},M_{j})$ is high only when $M_{i}$ and $M_{j}$ have comparable entropies ($B_{ij}$ is near 1) and significant structural difference ($\Delta_{ij}$ is large). If either condition fails (one view dominates in entropy or the two views are nearly identical), the product ensures $G(M_{i},M_{j})$ is small. This multiplicative form naturally enforces a “both-or-none” threshold: both entropy balance and structural complementarity must be present for a large gain. It is worth noting that no additional parameters are needed in this metric.

Next, we extend the pairwise gain to an entire group (subset) of views. For any subset $S\subseteq \{M_{1},\ldots ,M_{n}\}$ of size |S|=k, we define its group gain G(S) as the average of all pairwise gains within the group:

(9) G(S)=1k2∑Mi,Mj∈Si<jG(Mi,Mj).

This average pairwise gain is the group quality score. The mean over k2 pairs normalizes for group size. High G(S) indicates complementary structure and balanced information. A weak member lowers many pair scores and reduces the average. Hence G(S) is a reliable indicator of fusion value. By maximizing the overall group gain G(S) to adaptively identify the view subset corresponding to the optimal integration strategy, this module achieves a balanced and complementary fusion, thereby facilitating effective collaboration among views. The specific implementation of the optimized fusion mechanism module follows the steps outlined in Algorithm 2.

### 3.5. Model Details

Our framework is compatible with a wide range of graph neural network models, imposing no architectural limitations. In this study, to keep the design concise and to avoid oversmoothing effects commonly observed in deeper GCN architectures, we adopt a standard one-layer Graph Convolutional Network [37] as the basic encoder. A single layer also enables a fair and stable comparison across views, ensuring that the influence of multi-layer propagation does not obscure the effect of the proposed filtering and fusion mechanisms. Moreover, using a lightweight backbone allows us to isolate and more clearly evaluate the contribution of ViFi itself rather than the choice of encoder.

(10) $$g\left(\cdot \right):R^{N\times F}\times R^{N\times N}\to R^{N\times F_{h}}.$$

Its propagation rule is:

(11) $$g\left(X,A\right)=\sigma \;\left(\tilde{A}\;X\;\Theta \right),$$

where σ(·) is a standard nonlinearity (e.g., ReLU, softmax, sigmoid, or tanh). The symmetrically normalized adjacency matrix is defined as:

(12) $$\tilde{A}={\hat{D}}^{-\frac{1}{2}}\hat{A}{\hat{D}}^{-\frac{1}{2}},\;\hat{A}=A+I,\;\hat{D}=D+I.$$

Here, $A\in R^{N\times N}$ denotes the adjacency matrix encoding the graph connectivity, and $X\in R^{N\times F}$ represents the node feature matrix, where *N* is the number of nodes and *F* is the feature dimension. The identity matrix I is introduced to incorporate self-loops for each node, while D denotes the degree matrix of A. Accordingly, $\hat{D}$ corresponds to the degree matrix associated with $\hat{A}$.

**Algorithm 2** Optimized fusion mechanism**Input:** Filtered view set $\left\{M_{1},M_{2},\ldots ,M_{m}\right\}$; GNN encoder g(·); group size constraint k≥2; **Output:** Fused representation $\tilde{Y}$.1:**for** each pair of views $\left(M_{i},M_{j}\right)$ **do**2:   Compute entropy balance $B_{ij}$ and normalized structural difference $\Delta_{ij}$ via Equations (5) and (6);3:   Determine the pairwise gain $G(M_{i},M_{j})$ by multiplying the entropy balance and structural difference metrics to assess complementarity via Equation (8);4:**end for**5:**for** each candidate subset $S\subseteq \{M_{1},\ldots ,M_{m}\}$ with |S|=k **do**6:   Compute the group gain by averaging all pairwise gains within the subset to evaluate overall fusion potential via Equation (9);7:**end for**;8:Select the subset as optimal view integration strategy by identifying the candidate with the highest group gain value;9:Fuse the subset using g(·) and attention mechanism to integrate their representations;10:**Return** $\tilde{Y}$.

The trainable parameter matrix $\Theta \in R^{F\times F_{h}}$ maps the *F*-dimensional input features to $F_{h}$-dimensional hidden representations. Symmetric normalization rescales messages according to node degrees, which helps stabilize feature propagation during training. This single-layer formulation aggregates information from one-hop neighbors; however, the proposed fusion framework is not restricted to this choice and can be readily extended to other GNN backbones without altering the overall fusion pipeline.

**Local and global view encoders.** Each view encoder is composed of a GCN-based encoder together with its corresponding set of input views. These inputs may consist of different combinations of views, selected according to the chosen integration strategy. Rather than operating on a single view as in a typical GCN, both the local and global encoders receive multiple views simultaneously and generate their representations by applying mean pooling to the aggregated features:

(13) $$g_{\Theta_{\theta }}=\frac{1}{k}\sum_{G_{j}\in \left\{G_{1},G_{2},...,G_{k}\right\}}ReLU\left(\tilde{A}X_{j}\Theta_{\theta }\right).$$

**Attention mechanism.** To merge embeddings from the local and global encoders, we use an attention module to obtain a richer semantic representation.

Given *m* embeddings $H_{1},H_{2},\ldots ,H_{m}$, the attention module scores their contributions:

(14) $$\left(\alpha_{1},\alpha_{2},\ldots ,\alpha_{m}\right)=att\left(H_{1},H_{2},\ldots ,H_{m}\right).$$

Here $\alpha_{i}\in R^{N\times 1}$ gives node-wise weights for $H_{i}$. The coefficients follow $\alpha_{ij}=softmax\left(w_{ij}\right)$, with $w_{ij}=q_{i}^{T}\cdot \tanh\left(\Theta_{att}\cdot {\left(h_{j}\right)}^{T}+b_{i}\right)$, where $q_{i}\in R^{F^{\prime }\times 1}$ is a shared attention vector, $\Theta_{att}\in R^{F^{h}\times F^{\prime }}$ is the weight matrix, and $b_{i}\in R^{F^{\prime }\times 1}$ is the bias.

The fused embedding is the attention-weighted sum:

(15) $$H=\alpha_{1}\cdot H_{1}+\alpha_{2}\cdot H_{2}+\ldots +\alpha_{m}\cdot H_{m}.$$

**Figure 4:**
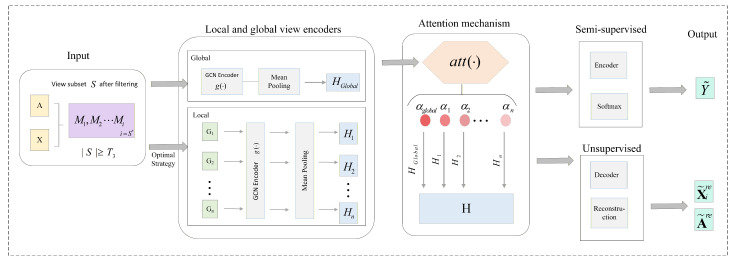
Detailed architecture of ViFi with semi-supervised and unsupervised learning settings.

In many real-world graph-based applications, obtaining labeled data for all nodes is often expensive or infeasible. Semi-supervised learning allows the model to leverage a limited set of labeled nodes together with the abundant unlabeled nodes, effectively improving generalization while reducing annotation costs. On the other hand, unsupervised learning architectures can exploit the inherent structural and feature information of graphs without relying on any labels, which is particularly useful when labels are entirely unavailable or scarce. Considering both semi-supervised and unsupervised settings enables the proposed framework to be versatile across different scenarios, ensuring robust performance whether partial labels are present or not. Motivated by these considerations, the following subsections present the details of the semi-supervised and unsupervised architectures adopted in the proposed framework. Figure 4 illustrates the detailed architecture of ViFi, which integrates both semi-supervised and unsupervised learning components within the proposed framework.

#### 3.5.1. Semi-Supervised Learning Architecture

In settings where only partial labels are available, typified by node classification, the framework concludes its representation extraction stage with a single-layer GCN that functions as the terminal encoder. The fused embedding H is subsequently processed by a single-layer Graph Convolutional Network, which functions as the final stage of encoding and generates the model’s representations $\tilde{Y}$:

(16) $$\tilde{Y}=softmax\left(\tilde{A}H\Theta_{final}\right).$$

#### 3.5.2. Unsupervised Learning Architecture

In unsupervised settings, incorporating a decoder enables the model to exploit self-supervised signals from the input graphs [38]. ViFi comprises a suite of encoders that capture complementary perspectives, whose outputs are aggregated into a unified embedding. Reconstruction is then achieved by pairing this fused representation with a set of decoders, each tasked with reconstituting a specific input view. Beyond the direct correspondence between input and output, an auxiliary decoder is incorporated to reconstruct the structural information of the original view. This reconstruction module is built from multiple two-layer components, with each layer crafted to serve as an approximate inverse of the encoder it mirrors.

The decoding stage produces two reconstructions: the node-feature matrix ${\tilde{X}}_{i}$ and the recovered view topology ${\tilde{A}}_{re}$. For the decoder associated with the *i*-th input view, the reconstructed node attributes are computed as:

(17) $${\tilde{X}}_{i}^{re}=softmax\left(\tilde{A}\;ReLU\left(\tilde{A}H\Theta^{\left(0\right)}\theta_{i}^{\prime }\right)\Theta^{\left(1\right)}\theta_{i}^{\prime }\right),$$

where $\Theta^{\left(l\right)}\theta_{i}^{\prime }$ represents the learnable weights associated with the *i*-th view-specific reconstruction decoder at layer *l*.

The output of the decoder is:

(18) $$\tilde{H}=\tilde{A}\;ReLU\left(\tilde{A}H\Theta_{\theta^{\prime }}^{\left(0\right)}\right)\Theta_{\theta^{\prime }}^{\left(1\right)},\;{\tilde{A}}^{re}=sigmoid\left(\tilde{H}{\tilde{H}}^{T}\right).$$

### 3.6. Training

The training objective of ViFi integrates two core terms: a complementary-learning objective and object loss [36]. The former, denoted as ($L_{c}$), aims to merge information drawn from multiple encoder viewpoints, whereas the latter, ($L_{o}$), drives the model to perform well on the specific downstream application.

We introduce a global loss function denoted as L to governs the entire training process:

(19) $$L=\eta \cdot L_{c}+\left(1-\eta \right)\cdot L_{o}.$$

**Discriminator and complementary loss.** To mitigate the issue that the global encoder alone may struggle to learn an optimal representation, a local encoder is incorporated to provide additional corrective signals. This design encourages the two encoding pathways to capture mutually informative cues, and the interaction between them is strengthened through a graph-based contrastive learning scheme. On this basis, the complementary objective ($L_{c}$) [36] is defined as follows:

(20) $$L_{c}=exp\left(-\frac{1}{\left|𝒱\right|}\sum_{l\in V}\sum_{i=1}^{m-1}\sum_{j\neq i}^{m}{∥Z_{l,i}-Z_{l,j}∥}_{2}\right).$$

where |𝒱| denotes the total number of nodes, and $Z_{l,i}$ (1<i≤m) represents the feature embedding of node *l* produced by the *i*-th view encoder.

**Object loss for semi-supervised and unsupervised learning.** For the semi-supervised setting, node classification is optimized by applying a cross-entropy objective to the embeddings corresponding to the labeled nodes:

(21) $$L_{o}=-\sum_{v_{k}\in {𝒱}_{L}}\sum_{j}^{C}Y_{kj}^{L}\log\left({\tilde{Y}}_{kj}\right).$$

In the unsupervised setting, the model relies on a combination of reconstruction loss and contrastive loss to realize self-supervised training. Accordingly, the task-specific loss for this scenario is formulated as follows:

(22) $$\begin{array}{c} L_{o}=\sum_{j\in \{G_{1},G_{2},...,G_{k}\}}{∥{\tilde{X}}_{j}-{\tilde{X}}_{j}^{re}∥}_{2}\\ +\lambda_{1}{∥\tilde{A}-{\tilde{A}}^{re}∥}_{2}+\lambda_{2}\sum_{i}^{m}\sum_{j\in Gset_{i}}\sum_{k\in Gset_{i},j\neq k}I_{j,k}^{i}. \end{array}$$

where $\lambda_{1}$ and $\lambda_{2}$ are hyperparameters. $Gset_{i}$ is the input set for the *i*-th view encoder.

### 3.7. Computational Complexity Analysis

To provide a clearer understanding of the computational requirements of ViFi, we present a brief analysis of the time and space complexity of its main components. The entropy-based view evaluation computes feature- and structure-related statistics for each view, resulting in a time complexity of O(m·(|V|+|E|)), where *m* denotes the number of views, and |V| and |E| represent the numbers of nodes and edges, respectively. Sorting the views according to their entropy scores introduces an additional cost of O(mlogm).

The subset exploration in the fusion stage has a theoretical worst-case complexity of $O(2^{m})$ when all possible view combinations are considered. However, in practice, the number of candidate views retained after the filtering step is small (typically m≤5 in our experiments), which significantly reduces the effective computational burden and avoids exhaustive search. In terms of space complexity, ViFi only maintains per-view representations and a limited number of candidate subsets during fusion, resulting in modest memory usage. Overall, the practical computational cost of ViFi remains low, and empirical runtime results show that the proposed framework introduces negligible overhead compared with standard multi-view GNN baselines.

## 4. Experiments

We conducted extensive experiments to validate the superiority of ViFi and answer the following research questions.

***Q1***: What is the performance of ViFi in node classification and graph classification tasks?
***Q2***: How can we verify the advantage of the ViFi framework?
***Q3***: What roles are fulfilled by the individual components within the proposed framework?
***Q4***: In what ways is the robustness of ViFi demonstrated?
***Q5***: How effective is the approach when it is utilized for node-clustering applications?
***Q6***: To what extent do changes in hyperparameter settings influence the behavior of ViFi?

**Datasets:** We conducted our evaluation on six benchmark datasets, whose main statistics are listed in Table 2. Cora, Citeseer, Pubmed, and DBLP are citation networks, whereas ACM and Chameleon were obtained from academic and Wikipedia sources, respectively.

**ViFi:** In the experimental analysis, **OURS** denotes the semi-supervised variant of ViFi, whereas **OURS-UN** refers to its unsupervised counterpart. For the unsupervised setting, the learned representations are subsequently fed into linear classifiers, allowing the model to produce node-classification results.

**Baselines:** We compared ViFi with state-of-the-art methods: (1) Base encoder: GCN [37]. (2) Attention-based encoders: GAT [39], MAGCN [40], DGCN [41], and PA-GCN [42]. (3) Multi-view information fusion-based encoders for node classification: MixHop [43], N-GCN [44], MOGCN [45], MAGCN [40], DGCN [41], PA-GCN [42], LoGo-GNN [36], StrucGCN [46], and ND-GCN [47]. (4) Multi-view information fusion-based encoders for graph classification: Co-GCN [48], LGCN-FF [49], SLFNet [50], HGCN-MVSC [51], and MGCN-DNS [52]. (5) Contrastive learning-based encoders: NCLA [53], PA-GCN [42], GraphCL [54], IGCL [55], and GCA [56]. (6) Unsupervised learning models: K-means, Deepwalk [57], GAE [38], and VGAE [38].

**Parameter Settings:** A complete list of hyperparameter settings for each dataset is provided in Table 3.

**Implementation Details:** A full-batch strategy was adopted for each epoch in our training procedure. The method was implemented in Pytorch, and parameter updates were carried out using the Adam [58] algorithm. For the standard graph benchmarks, we randomly sampled different numbers of labeled nodes per class for training while keeping 1000 nodes fixed for testing. In superpixel datasets, evaluation was conducted on a set of 10,000 images. All classification accuracy (ACC) values were averaged over 10 independent runs using the data splits described above. The hyperparameter η was explored across {0.05, 0.1, 0.15, …, 0.95}, while $\lambda_{1}$ and $\lambda_{2}$ were adjusted within {0, 0.1, 0.2, …, 1}. Additionally, the cosine threshold was tuned in {0.1, 0.15, 0.2, …, 0.5}, and *k* was varied over {5, 10, 15, …, 30}. The final results for each dataset are reported using the hyperparameter combination and iteration count that yield optimal performance.

**Evaluation Metrics:** Following established practices in node and graph classification, we evaluated the performance of both baseline methods and ViFi on node classification tasks using classification accuracy (ACC). For each dataset, ACC was computed across all test samples. In addition, to assess clustering effectiveness, we employed normalized mutual information (NMI) [59] and the adjusted rand index (ARI) [60], providing complementary measures of how well ViFi and competing approaches capture underlying cluster structures.

### 4.1. Performance on Node and Graph Classification (Q1)

#### 4.1.1. Performance on Node Classification

This section reports the average classification accuracy (ACC) along with its standard deviation across 10 independent trials. For reference, the results of DeepWalk [57], NCLA [53], LGCN-FF [49], and SLFNet [50] were adopted from their respective original studies. The outcomes of semi-supervised node classification are compiled in Table 4, with the key insights summarized as follows:

1. Compared with the baseline models, ViFi demonstrates consistently superior performance across most datasets. Notably, the semi-supervised version of ViFi (**OURS**) achieves better results than its unsupervised counterpart (**OURS-UN**). This improvement can be explained by the fact that the semi-supervised framework of ViFi adopts an end-to-end fusion mechanism, in which the available label information effectively guides and refines the embedding fusion process during model training.
2. The semi-supervised version of ViFi (**OURS**) consistently outperforms other models that incorporate multi-topology or multi-view information fusion, such as MAGCN, MOGCN, and PA-GCN. Moreover, the unsupervised version of ViFi (**OURS-UN**) also achieves better performance than most graph contrastive learning methods, including GCA and IGCL. This advantage primarily stems from ViFi’s ability to identify and retain only the views that contribute positively to representation while filtering out irrelevant views that contribute negatively, thereby enhancing representation quality and improving the model’s robustness against noisy or irrelevant information.
3. Building on the failure cases listed in Table 4, we selected the Pubmed dataset as a representative example for a more detailed analysis. In this dataset, the performance of ViFi is inferior to that of the NCLA model. This phenomenon can be attributed to a key characteristic of NCLA: it employs an augmentation-based learning strategy, which facilitates effective extraction of self-supervised signals between the original graph and its augmented versions, provided that the underlying relationships in the raw graph are trustworthy. A comparable pattern emerges when examining the Citeseer dataset. In this case, ViFi also trails behind the LA-GCN model. This performance gap can be attributed to the design of LA-GCN, which incorporates a trainable local augmentation mechanism grounded in the structural relations of the original graph. Meanwhile, it places greater emphasis on enhancing the informative value of data from the perspective of feature engineering, which further explains its performance advantage over ViFi in this dataset. However, overall, ViFi continues to exhibit better performance compared with these competing approaches. The superior performance of ViFi can be mainly attributed to its entropy-driven adaptive view filter and optimized fusion mechanism. The adaptive view filter evaluates the similarity and connectivity among nodes to characterize the concentration of information distribution. Based on this, it retains the views that contain richer and more informative content for subsequent fusion. In the optimized fusion mechanism, a novel information gain function is proposed to evaluate candidate view groupings based on entropy balance and structural complementarity. It is further employed to determine the most effective integration strategy, thereby enabling a more efficient and complementary fusion of multi-view representations. The superiority of ViFi becomes particularly pronounced in the presence of noise. The following experimental section examines and quantifies the robustness of the model.

#### 4.1.2. Performance on Graph Classification

We used the multi view datasets for graph classification and took GCN-fusion [37], Co-GCN [48], LGCN-FF [49], SLFNet [50], HGCN-MVSC [51], and MGCN-DNS [52] as baselines. The reported classification accuracy (ACC) represents the average over 10 independent runs. Graph classification outcomes are summarized in Table 5, demonstrating that ViFi maintains strong competitiveness in graph-level classification tasks.

### 4.2. ViFi Architecture Study (Q2)

To simplify the analysis, we employed only the best-performing variant of ViFi (OURS) built upon a semi-supervised learning framework. The model was applied to node classification and semantic similarity tasks on the Cora dataset, providing additional validation for the effectiveness of the proposed entropy-based adaptive view filtering and the optimized fusion strategy.

- Multi-view GCN+MLP: A GCN framework combining local and global encoder views via MLP was utilized to perform node classification tasks;
- Multi-view GAT+GAT: A graph attention network (GAT) architecture that merges local and global encoder views using a single-layer GAT was employed for node level classification tasks;
- Global-GCN+GCN: A GCN architecture adopting a global encoder perspective, implemented with a single-layer GCN for node classification tasks;
- **OURS-UN**-w/o: ViFi implemented within an unsupervised learning framework, operating without the complementary loss term $L_{c}$;
- **OURS**-w/o: ViFi implemented within a semi-supervised learning framework, operating without the complementary loss term $L_{c}$;
- **OURS-UN**: ViFi configured within an unsupervised learning framework;
- **OURS**: ViFi configured within a semi-supervised learning framework;

These results lead to several noteworthy observations, as discussed below:

1. To further assess the efficacy of the ViFi framework, we utilized a standard MLP or a graph attention network as the terminal classifier. The experimental results indicate that incorporating the graph attention network (GAT) yields superior performance compared with both the MLP-based and our proposed configurations. This improvement suggests that the GAT provides a more efficient mechanism for capturing the topological dependencies within the fused node representations. Moreover, the performance of our model surpasses that of the multi-view GCN+MLP, implying that the fusion process preserves the essential relational patterns among nodes.
2. Compared with the Global-GCN+GCN variant, ViFi delivers superior results, underscoring the necessity of incorporating localized structural cues when global information is sparse or partially missing, as is often the case under limited label supervision. Furthermore, architectures that jointly exploit global and local views tend to exhibit more stable and effective behavior than approaches relying solely on a global-view encoder.
3. Acquiring complementary information from different views plays a vital role. As shown in Table 6, the ViFi (**OURS-UN** and **OURS**) trained with the global loss function achieves better performance than the ViFi trained solely with the object loss $L_{o}$. This observation further validates our rationale for introducing the global loss function.

### 4.3. Ablation Study (Q3)

The effectiveness of the proposed ViFi is confirmed through the comparative experiments discussed above. In addition, to further validate the contribution of each individual component within ViFi, we conducted a series of ablation studies.

#### 4.3.1. Entropy-Based Adaptive View Filter

First, we applied various aggregation strategies to generate augmented graphs, each serving as a distinct view. For comparison, we incorporated the GCN aggregation method [37] into the graph augmentation process (Equation (23)). Table 7 summarizes the descriptions of the various views. To visualize the representational capacity of each view (PR, PC, RawC, and Raw), a broken-line chart was employed, tracking changes after each iteration. The alignment between each augmented view and the original input view (Raw) was subsequently quantified by examining their semantic similarity. Both the augmented and original views were encoded using a shared GCN encoder with identical parameter settings, and their learned graph representations were subsequently compared to assess semantic alignment.

(23) $${\tilde{x}}_{i}={\hat{A}}_{i,i}x_{i}+\sum_{x_{j}\in N\left(x_{i}\right)}{\hat{A}}_{i,j}x_{j}.$$

The results in Table 8 indicate that the performance of augmented views varies notably across datasets, reflecting the distinct structural and semantic characteristics captured by each view. The PC view attains the highest accuracy on the Cora and ACM datasets, suggesting that cosine similarity preserves meaningful feature correlations beneficial for classification, while the PK view performs slightly better on Pubmed, implying that neighborhood-based relations are more informative in this dataset. Such inconsistency among single views highlights the necessity of filtering and integrating multiple complementary views. When multiple views are combined, classification accuracy improves substantially compared with individual views. Among them, Raw+PC+PK consistently achieves the best results across all datasets, indicating that combining feature-similarity and structural-proximity views enhances the representational capacity of the model and leads to more effective multi-view learning.

**Figure 5:**
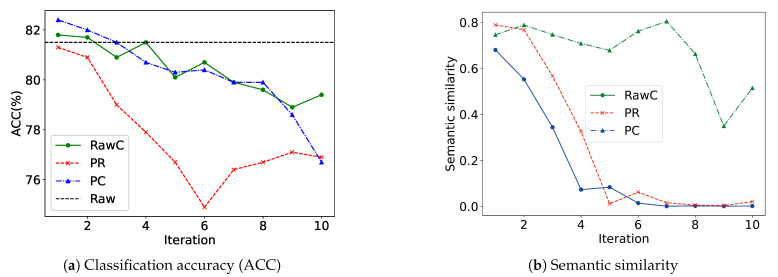
Comparison of classification accuracy and semantic similarity across iterations for RawC, PR, and PC.

The analysis of semantic similarity and representation ability of Figure 5 provides a key explanation for this phenomenon. The figure shows that the semantic similarity and representation ability between different views show different evolution trajectories with the change in the number of iterations. The PC view can obtain higher representation ability with fewer iterations and maintain a moderate semantic distance from the original view, which can provide effective complementary information, but the ability to improve is limited. This inconsistency in performance and properties implies that directly fusing all views without any filtering mechanism may introduce interfering or task-irrelevant views, thereby degrading model performance. Moreover, the model would fail to adaptively identify the most informative views for the given task, ultimately resulting in suboptimal fusion outcomes. Therefore, a filtering mechanism that evaluates and adaptively filters views is essential to ensure that the fusion process focuses on the most informative and complementary views.

#### 4.3.2. Optimized Fusion Mechanism

Furthermore, we designed a series of comparative experiments to investigate the role of the optimized fusion mechanism. Specifically, we implemented several fusion variants, including manual fusion, random subset selection, and simplified versions, that retain only the entropy-balance or structural-difference term. Each variant employs the same set of filtered views obtained from the first module, ensuring that the observed differences arise solely from the fusion mechanism. The descriptions of these variants are summarized in Table 7. Subsequently, we compared their classification accuracy across multiple datasets to evaluate the impact of different fusion mechanisms.

The experimental results presented in Table 9 reveal several important observations. The manual fusion setting yields the lowest accuracy across all datasets, indicating that fixed integration schemes fail to capture the complementary information among views effectively. The random selection strategy shows only marginal improvement, suggesting that arbitrary integrations of views cannot guarantee consistent information gain. The variants using only the entropy-balance or only the structural-difference term both lead to moderate performance gains, implying that each component contributes partially to the overall fusion objective. The greedy-by-entropy approach achieves slightly higher accuracy, demonstrating that prioritizing informative views provides a limited but noticeable benefit. The fully optimized fusion mechanism achieves the highest accuracy across datasets, confirming that jointly considering entropy balance and structural complementarity enables more adaptive and synergistic view integration. Overall, the results validate the necessity of automatic strategy selection in promoting balanced and effective multi-view fusion.

### 4.4. Robustness Analysis (Q4)

As both the unsupervised (**OURS-UN**) and semi-supervised (OURS) variants of VF-GRFN employ an identical fusion framework, only the semi-supervised model (OURS), which demonstrates superior performance, was utilized in the subsequent experiments to streamline the analysis.

**Figure 6:**
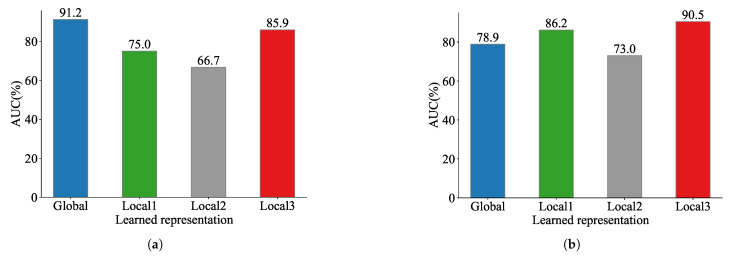
VGAE graph reconstruction performance on Cora across views. (**a**) AUC for unattacked inputs (learned from original views). (**b**) AUC for attacked inputs (learned from views with 80% edges randomly deleted).

**Figure 7:**
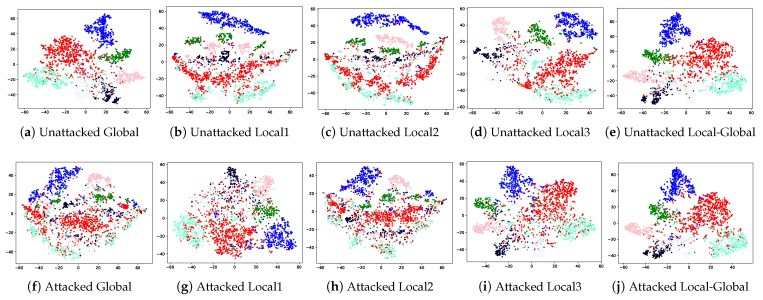
Visualization of learned embeddings on Cora across views. Unattacked (**a**–**e**): from original views. Attacked (**f**–**j**): from views with 80% edges randomly deleted.

First, we evaluated the expressive capability of each view through visualization and graph reconstruction techniques. To accomplish the reconstruction of the latent representations derived from each view-specific encoder on the Cora dataset, we utilized a Variational Graph Autoencoder (VGAE) [38]. The reconstruction quality was quantified using the AUC (area under the ROC curve) metric, and the corresponding results are illustrated in Figure 6. Furthermore, to intuitively demonstrate the distribution of the learned representations, we visualize the embeddings of each view encoder using t-SNE [62], as shown in Figure 7.

**Figure 8:**
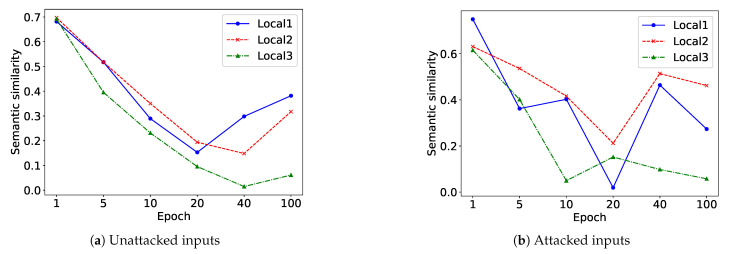
Semantic similarity between local and global view embeddings on Cora. (**a**) Unattacked inputs (from original views). (**b**) Attacked inputs (from views with 80% edges randomly removed).

On the Cora benchmark, we monitored the evolution of semantic similarity across embeddings from the global and local views throughout the learning process. This is presented through a line-based visualization computed at selected training epochs {1; 5; 10; 20; 40; 100}. The embedding vectors for all views were obtained through the same GCN encoder. Their semantic similarity was subsequently quantified by analyzing the resulting feature representations, as depicted in Figure 8. Table 7 shows a description of various views. When the input graphs are perturbed, the expressive capability of each view encoder declines as both its visual distribution and its reconstruction quality deteriorate. In contrast, the fused representation that integrates local and global views preserves stronger expressiveness under noisy conditions, indicating that ViFi is more robust. The global-only representation performs less effectively, while local views exhibit higher discriminability and better adaptation to structural noise. Moreover, the semantic discrepancy between local and global representations grows with the proposed contrastive loss $L_{c}$, confirming the significance of introducing local views. Our objective is enabled to enhance diversity among encoders from different views while maintaining the strong learning ability of key encoders. However, this divergence does not continually increase with training epochs as the optimization is jointly constrained by contrastive and objective losses.

**Figure 9:**
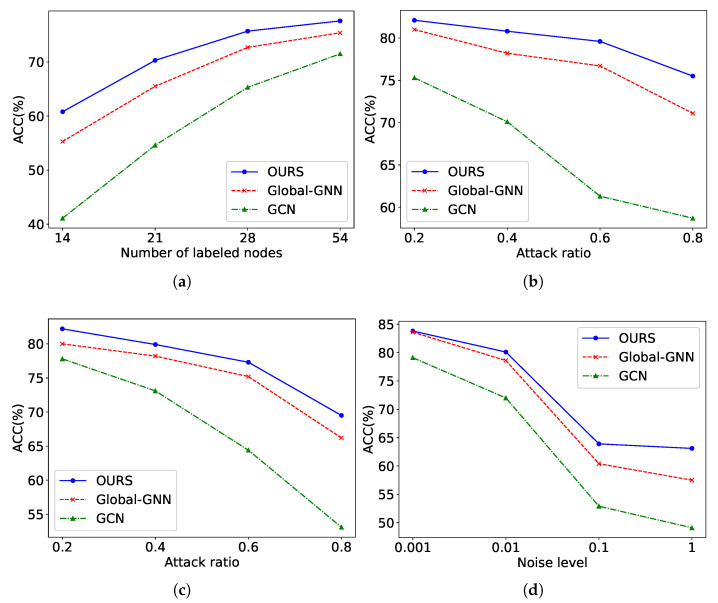
Performance Comparison of GCN, Global-GNN, and **OURS** on Cora. (**a**) Different label rates. (**b**) Random topology attack. (**c**) Random feature mask. (**d**) Node noise attack.

To assess the robustness of ViFi, we further subjected ViFi and its variants Global-GNN and GCN [37] to a set of four uncertainty scenarios. These conditions were used to examine how reliably each model maintains classification performance when exposed to varying views. These uncertainties may introduce disturbances to the graph structure or node semantics, thereby affecting classification accuracy. All experiments were conducted on the Cora dataset. The number of labeled nodes was set to {14; 21; 28; 54}. The attack and mask ratios were {0.2; 0.4; 0.6; 0.8}, and the noise level was adjusted to {0.001; 0.01; 0.1; 1}. For topology and feature masking, we randomly removed a portion of edges or node features according to the specified ratio. The modified graphs were then used to test classification performance on the corrupted data. In the case of feature corruption caused by node noise, we perturbed the input attributes by injecting Gaussian disturbances, where the parameter noise level controls the strength of the added variation. This process intentionally distorts the original feature distribution to evaluate model stability with corrupted inputs. The corresponding outcomes are presented in Figure 9. Drawing from these observations, several key findings can be outlined as follows.

1. As illustrated in Figure 9a, the baseline model experiences a sharp decline in performance as the label rate decreases. In contrast, OURS maintains superior accuracy even under extremely low label availability conditions. This observation indicates that the introduction of a view filter and an optimized fusion mechanism enhances the model’s ability to capture essential features under limited supervision. Notably, OURS substantially outperforms Global-GNN in such low-label scenarios, further validating the effectiveness of the proposed design.
2. As illustrated in Figure 9b, **OURS** consistently achieves superior performance compared to the baseline models with higher attack ratios. This advantage arises from its ability to effectively capture information from the entropy-based view filter. In general, all methods exhibit a sharp decline in performance as the random attack ratio increases.
3. In alignment with earlier findings, the results presented in Figure 9c,d indicate that **OURS** maintains a clear advantage over both GCN and Global-GNN across varying noise and masking conditions. Overall, as the proportion of masked features or the strength of the injected noise increases, every method exhibits a substantial reduction in predictive accuracy.

### 4.5. Performance on Node Clustering (Q5)

We further examined the capability of the framework in unsupervised graph representation learning. Once the model was trained, the fused node representations $H^{\left(1\right)}$ were extracted and subsequently grouped using the K-means clustering procedure on three benchmark datasets—Cora, Citeseer, and Pubmed. The evaluation was based on two metrics, normalized mutual information (NMI) and the adjusted rand index (ARI), and their mean values along with standard errors are reported in Table 10. To establish competitive baselines, we incorporated a range of widely used unsupervised and contrastive graph learning methods, namely, K-means, DeepWalk [57], GraphCL [54], IGCL [55], GCA [56], GAE [38], and VGAE [38]. To further illustrate the flexibility of the ViFi framework, the objective $L_{o}$ was equipped with alternative contrastive components by substituting its I term with the loss formulations of GCA [56], IGCL [55], and GraphCL [54]. These variants are referred to as OURS, OURS(GraphCL), and OURS(IGCL), respectively. The experimental results indicate that ViFi achieves performance comparable to other state-of-the-art methods across most datasets. These results confirm that ViFi exhibits strong competitiveness in unsupervised clustering tasks. Furthermore, the model’s performance is highly sensitive to the choice of I loss, underscoring its inherent flexibility.

### 4.6. Parameter Sensitivity (Q6)

Sensitivity studies were conducted on the key hyperparameters to examine their influence on model performance. ViFi was trained with α values ranging from 0.05 to 0.95, with an interval of 0.05. Experimental observations indicate that α values between 0.3 and 0.7 produce the most favorable outcomes, suggesting that maintaining a balanced emphasis between feature entropy and structural entropy enables the model to effectively capture both semantic richness and topological diversity, thereby achieving more comprehensive and discriminative representations. ViFi was trained with μ values ranging from 0.01 to 0.5, with an interval of 0.01. The experimental results show that moderate μ values, typically between 0.1 and 0.3, yield the best performance, suggesting that an appropriate regularization strength helps balance the trade-off between maximizing information gain and controlling the size of selected view subsets, thereby preventing overfitting to irrelevant views while maintaining informative diversity. We trained ViFi with η values from 0.05 to 0.95 and observe that 0.4–0.8 yields the best performance, indicating that appropriately weighting cross-encoder representation relationships improves the model.

**Figure 10:**
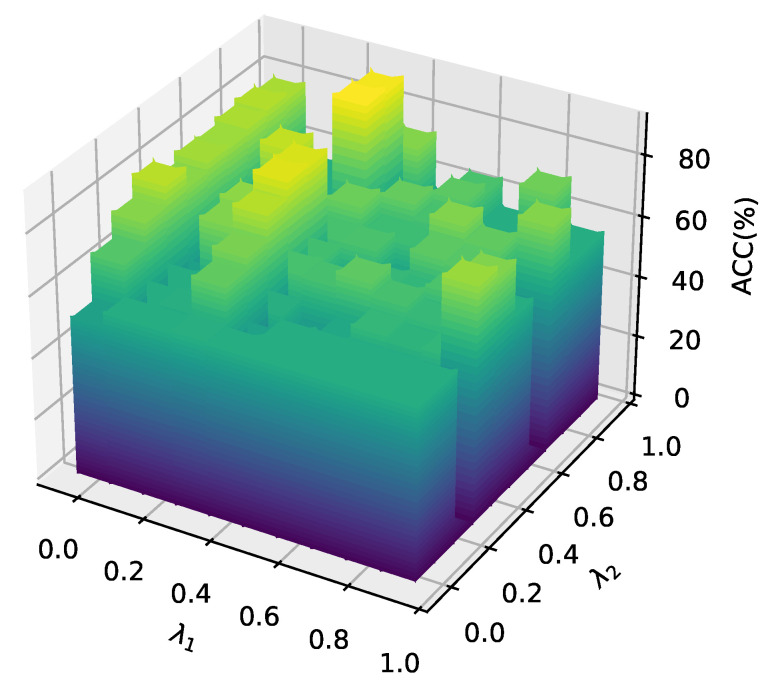
Impact of hyperparameters $\lambda_{1}$ and $\lambda_{2}$ on **OURS-UN**’s node classification accuracy (ACC%).

Furthermore, we investigated the effects of the hyperparameters $\lambda_{1}$ and $\lambda_{2}$ on the model’s performance. In this experiment, other hyperparameters were fixed while $\lambda_{1}$ and $\lambda_{2}$ varied from 0 to 0.9. The classification results of all nodes are visualized in a 3D bar chart, as shown in Figure 10. The findings reveal that higher values of $\lambda_{1}$ and $\lambda_{2}$ generally lead to improved self-supervised learning performance.

## 5. Conclusions

In this paper, we proposed a novel multi-view representation learning framework called the view filter-driven graph representation fusion network (ViFi). Following the “less for better” principle, ViFi aims to obtain more effective graph representations by leveraging fewer but more informative views. The framework first evaluates the feature–topology entropy of each view to measure its information quality, adaptively filtering those that provide complementary signals. It then integrates the filtered views through an information gain function that balances information diversity and structural consistency, ensuring collaborative and complementary fusion. Extensive experiments on classification and clustering tasks verified the effectiveness and superior performance of the proposed ViFi.

Despite the promising results, ViFi still has several limitations. The current entropy estimation relies on predefined statistical formulations, which may not fully capture higher-order correlations or latent semantic dependencies among views. Moreover, the fusion process is optimized based on pairwise information gain, potentially overlooking more complex multi-view interactions that could further enhance representation quality. Future work will explore learnable entropy modeling and more flexible fusion mechanisms to better capture inter-view dependencies and improve the generalization of the framework.

## Figures and Tables

**Table 1:** Notation description for formulas.

Notation	Description
G	A graph
𝒱	Node set
E	Edge set
A	Adjacency matrix
X	Node feature matrix
$M_{i}$	*i*-th graph view
*S*	Selected subset of views
$H_{f}\left(M_{i}\right)$	Feature entropy of view $M_{i}$
$H_{s}\left(M_{i}\right)$	Topology entropy of view $M_{i}$
$I(M_{i})$	View information score
*k*	Order of optimal integration strategy
F(S)	Subset score
$T_{3}$	Minimum number of views
$B_{ij}$	Entropy balance
$\Delta_{ij}$	Normalized structural difference
$G(M_{i},M_{j})$	Pairwise gain
G(S)	Group gain
g(·)	GNN encoder
ϕ(·)	Size penalty function

**Table 2:** Data details.

Dataset	Nodes	Edges	Features	Classes	Training	Test
Cora	2708	5429	1433	7	140	1000
Citeseer	3327	4732	3703	6	120	1000
ACM	3025	13,128	1870	3	60	1000
Chameleon	2277	36,101	2325	4	80	1000
DBLP	17,716	105,734	1639	4	80	1000
Pubmed	19,717	44,338	500	3	60	1000
MNIST	—	—	—	10	55,000	10,000
ALOI	—	—	—	100	800	200
NUS-WIDE	—	—	—	81	161,789	107,859
MSRC-v1	—	—	—	7	105	200

**Table 3:** Hyperparameter settings.

Dataset	Learning Rate	Weight Decay	Training Epochs	Dropout
Cora	0.0015	5.00 × $10^{-4}$	200	0.3
Citeseer	0.0015	5.00 × $10^{-4}$	200	0.3
ACM	0.0015	5.00 × $10^{-4}$	200	0.3
Chameleon	0.0015	5.00 × $10^{-4}$	200	0.3
DBLP	0.0015	5.00 × $10^{-4}$	200	0.3
Pubmed	0.0015	5.00 × $10^{-4}$	200	0.3
MNIST	0.001	1.00 × $10^{-5}$	5000	0.5
ALOI	0.001	1.00 × $10^{-5}$	5000	0.5
NUS-WIDE	0.001	1.00 × $10^{-5}$	5000	0.5
MSRC-v1	0.001	1.00 × $10^{-5}$	5000	0.5

**Table 4:** The classification accuracy (ACC%) for node level tasks is reported, where **A** and **X** are the adjacency matrix and feature matrix, and **Y** is the label information (bold: best).

Method	Training Data	Datasets					
		Cora	Citeseer	ACM	Chameleon	DBLP	Pubmed
DeepWalk [57]	A	67.2	43.2	-	-	-	65.3
NCLA [53]	X,A	82.2	71.7	-	-	-	**82.0**
GCA [56]	X,A	82.7 ± 0.6	71.8 ± 0.7	89.4 ± 0.6	35.6 ± 0.2	70.6 ± 0.5	76.8 ± 0.5
IGCL [55]	X,A	83.5 ± 0.5	72.1 ± 0.6	89.2 ± 0.7	47.6 ± 0.4	73.6 ± 0.3	80.8 ± 0.6
GraphCL [54]	X,A	83.1 ± 0.6	72.1 ± 0.6	90.2 ± 0.6	48.6 ± 0.5	72.9 ± 0.5	80.6 ± 0.6
**OURS-UN**	X,A	83.5 ± 0.4	73.0 ± 0.3	91.6 ± 0.7	51.5 ± 0.6	73.8 ± 0.3	80.9 ± 0.8
GCN [37]	X,A,Y	81.5 ± 0.2	70.4 ± 0.4	87.8 ± 0.2	47.6 ± 0.4	70.2 ± 0.5	79.0 ± 0.6
GAT [39]	X,A,Y	83.2 ± 0.7	72.6 ± 0.7	87.4 ± 0.3	47.9 ± 0.4	71.0 ± 0.3	79.0 ± 0.6
PA-GCN [42]	X,A,Y	83.6 ± 0.2	70.4 ± 0.3	90.9 ± 0.3	49.0 ± 0.3	72.0 ± 0.4	79.3 ± 0.3
MOGCN [45]	X,A,Y	82.4 ± 1.2	72.4 ± 0.8	90.1 ± 1.4	46.9 ± 0.4	70.9 ± 0.7	79.2 ± 0.3
N-GCN [44]	X,A,Y	83.0 ± 0.5	72.2 ± 0.5	88.0 ± 0.3	48.9 ± 0.4	71.3 ± 0.2	79.5 ± 0.4
MixHop [43]	X,A,Y	81.9 ± 0.4	71.4 ± 0.6	87.9 ± 0.7	40.6 ± 0.6	70.9 ± 0.3	80.8 ± 0.2
DGCN [41]	X,A,Y	84.1 ± 0.3	73.3 ± 0.1	90.2 ± 0.2	48.9 ± 0.4	72.3 ± 0.2	80.2 ± 0.3
MAGCN [40]	X,A,Y	84.5 ± 0.5	73.3 ± 0.3	90.6 ± 0.3	50.4 ± 0.5	72.5 ± 0.3	80.6 ± 0.8
LA-GCN [61]	X,A,Y	84.6 ± 0.7	**74.7 ± 0.5**	89.9 ± 0.4	48.3 ± 0.6	72.0 ± 0.5	81.7 ± 0.7
LoGo-GNN [36]	X,A,Y	83.9 ± 0.4	73.4 ± 0.5	91.8 ± 0.5	51.7 ± 0.3	73.9 ± 0.4	81.6 ± 0.7
StrucGCN [46]	X,A,Y	82.8 ± 0.6	72.3 ± 0.4	90.7 ± 0.3	50.6 ± 0.5	73.1 ± 0.3	81.4 ± 0.4
ND-GCN [47]	X,A,Y	83.2 ± 0.4	73.6 ± 0.3	89.9 ± 0.6	51.2 ± 0.3	73.5 ± 0.4	80.8 ± 0.5
**OURS**	X,A,Y	**84.2 ± 0.4**	73.8 ± 0.5	**94.8 ± 0.5**	**52.7 ± 0.3**	**74.9 ± 0.5**	**83.6 ± 0.7**

**Table 5:** The classification accuracy (ACC%) for graph level tasks (bold: best).

Method	Datasets			
	ALOI	MNIST	NUS-WIDE	MSRC-v1
GCN-fusion [37]	89.4	86.6	36.7	58.5
Co-GCN [48]	90.2	90.8	26.5	67.2
LGCN-FF [49]	93.4	90.2	38.3	83.4
SLFNet [50]	91.7	95.6	45.7	76.1
HGCN-MVSC [51]	92.3	91.1	44.3	81.5
MGCN-DNS [52]	92.2	94.7	45.2	82.1
**OURS-UN**	93.4	95.2	47.3	85.2
**OURS**	**94.8**	**96.3**	**48.8**	**86.7**

**Table 6:** Classification accuracy (ACC%) for ViFi and its variant models (bold: best).

Method	Cora	Citeseer	ACM	Chameleon	DBLP	Pubmed
Multi views-GCN+MLP	84.2	72.4	90.1	49.5	72.1	80.3
Multi views-GAT+GAT	**84.7**	73.3	92.0	**52.9**	**75.2**	**83.6**
Global-GCN+GCN	84.5	73.2	91.6	51.7	74.5	81.2
**OURS-UN**-w/o	82.5	71.5	90.4	51.2	72.8	80.5
**OURS**-w/o	84.3	73.3	91.5	51.3	73.1	80.9
**OURS-UN**	83.5	71.7	91.6	51.5	73.8	80.9
**OURS**	84.2	**73.8**	**94.8**	52.7	74.9	**83.6**

**Table 7:** Notation description for experiments.

Notation	Description
Raw	The raw input view.
RawC	The convolution augmentation view via Equation (23).
PR	The augmented view based on raw view relations.
PC	The augmented view based on cosine similarity view relations.
PK	The augmented view based on *k*NN view relations.
Global	Learned representation of global views ({RAW, PC, PK}).
Local1	Learned representation of local views ({RAW, PC}).
Local2	Learned representation of local views ({RAW, PK}).
Local3	Learned representation of local views ({PC, PK}).
Local-Global	Fused representation from the local-to-global views.
No-Opt (manual)	Fusion without optimization; a fixed manual strategy is used to integrate filtered views.
Random-Select (avg)	Randomly selects view subsets of the same size for fusion and averages the results.
Only-Entropy	Employs only the entropy-balance term in the gain function.
Only-Structure	Employs only the normalized structural-difference term in the gain function.
Greedy-by-Entropy	Sequentially adds views based on their individual information scores until the gain ceases to improve.
Full (Ours)	Complete optimized fusion mechanism using both entropy balance and structural complementarity to adaptively select the optimal view integration.

**Table 8:** The accuracy (%) achieved by the GCN encoder when operating on various individual views or integration of views (bold: best).

Input	Cora	Citeseer	ACM	Chameleon	DBLP	Pubmed
RAW	81.2	70.6	87.5	47.9	70.4	78.8
PR	81.0	70.3	88.0	47.1	71.0	78.1
PC	**82.3**	**71.2**	**88.5**	**49.4**	**71.7**	80.0
PK	82.1	70.8	87.6	48.1	70.2	**80.4**
Raw + PR + PC	83.6	71.3	90.9	50.4	72.7	80.3
Raw + PR + PK	83.1	71.0	91.1	50.1	71.8	80.9
Raw + PC + PK	**84.2**	**73.8**	**94.8**	**52.7**	**74.9**	**83.6**

**Table 9:** Ablation study on the optimized fusion mechanism (ACC%) (bold: best).

Method	Cora	Citeseer	ACM	Chameleon	DBLP	Pubmed
No-Opt (manual)	83.7	71.5	91.0	50.6	72.9	80.4
Random-Select (avg)	83.9	71.9	91.2	51.0	73.2	80.7
Only-Entropy	84.0	72.1	91.4	51.4	73.6	81.0
Only-Structure	84.1	72.3	91.5	52.0	73.8	81.2
Greedy-by-Entropy	84.2	72.6	91.6	52.1	74.0	81.3
**Full (Ours)**	**84.2**	**73.8**	**94.8**	**52.7**	**74.9**	**83.6**

**Table 10:** Theclustering performance of various approaches with different input settings, where **A** and **X** are the adjacency matrix and feature matrix, and **Y** is the label information (bold: best).

Method	Training Data	Cora		Citeseer		Pubmed	
		NMI	ARI	NMI	ARI	NMI	ARI
K-mean	X	32.1	23.0	30.5	27.9	0.1	0.2
DeepWalk	X	32.7	24.3	8.8	9.2	27.9	29.9
GAE	X,A	42.9	34.7	17.6	12.4	27.7	27.9
VGAE	X,A	23.9	17.5	15.6	9.3	22.9	21.3
GRAGE	X,A	54.4 ± 1.2	43.4 ± 3.1	35.2 ± 1.8	34.1 ± 1.7	30.0 ± 3.4	29.5 ± 2.1
**OURS**	X,A	55.6 ± 2.3	47.2 ± 2.5	39.2 ± 2.3	35.1 ± 2.3	32.5 ± 2.6	33.7 ± 2.0
GraphCL	X,A	55.3 ± 3.2	54.5 ± 2.7	42.1 ± 3.9	44.1 ± 1.8	34.1 ± 3.3	34.2 ± 2.8
**OURS (GraphCL)**	X,A	56.9 ± 2.2	55.0 ± 2.3	45.0 ± 2.0	44.3 ± 2.1	35.6 ± 2.3	33.8 ± 1.5
IGCL	X,A	56.6 ± 3.0	53.9 ± 2.0	43.5 ± 1.7	**45.2 ± 1.9**	33.4 ± 2.6	32.9 ± 1.1
**OURS (IGCL)**	X,A	**57.9 ± 1.3**	**55.2 ± 2.2**	**45.2 ± 2.3**	44.8 ± 2.6	**37.5 ± 2.4**	**35.7 ± 1.8**

## Data Availability

The original contributions presented in this study are included in the article. Further inquiries can be directed to the corresponding author.
